# A Symmetric Dual Feedback System Provides a Robust and Entrainable Oscillator

**DOI:** 10.1371/journal.pone.0030489

**Published:** 2012-02-20

**Authors:** Kazuhiro Maeda, Hiroyuki Kurata

**Affiliations:** Department of Bioscience and Bioinformatics, Kyushu Institute of Technology, Iizuka, Fukuoka, Japan; Georgia State University, United States of America

## Abstract

Many organisms have evolved molecular clocks to anticipate daily changes in their environment. The molecular mechanisms by which the circadian clock network produces sustained cycles have extensively been studied and transcriptional-translational feedback loops are common structures to many organisms. Although a simple or single feedback loop is sufficient for sustained oscillations, circadian clocks implement multiple, complicated feedback loops. In general, different types of feedback loops are suggested to affect the robustness and entrainment of circadian rhythms.

To reveal the mechanism by which such a complex feedback system evolves, we quantify the robustness and light entrainment of four competing models: the single, semi-dual, dual, and redundant feedback models. To extract the global properties of those models, all plausible kinetic parameter sets that generate circadian oscillations are searched to characterize their oscillatory features. To efficiently perform such analyses, we used the two-phase search (TPS) method as a fast and non-biased search method and quasi-multiparameter sensitivity (QMPS) as a fast and exact measure of robustness to uncertainty of all kinetic parameters.

So far the redundant feedback model has been regarded as the most robust oscillator, but our extensive analysis corrects or overcomes this hypothesis. The dual feedback model, which is employed in biology, provides the most robust oscillator to multiple parameter perturbations within a cell and most readily entrains to a wide range of light-dark cycles. The kinetic symmetry between the dual loops and their coupling via a protein complex are found critically responsible for robust and entrainable oscillations. We first demonstrate how the dual feedback architecture with kinetic symmetry evolves out of many competing feedback systems.

## Introduction

Many organisms have evolved molecular clocks to anticipate daily changes in the environment [1], [2]. The molecular mechanisms by which the circadian clock network produces sustained cycles have extensively been studied and transcriptional-translational feedback loops are known as common structures to many organisms [1]. Although a simple or single feedback loop is sufficient for sustained oscillations [3], circadian clock systems implement complicated feedback loops. A current problem is to reveal the mechanism by which such a complex feedback system evolves.

Mathematical models for circadian clocks have been proposed and extensively studied [4]–[12]. In most studies, nominal or convenient values are assigned to kinetic parameters, because experimental data are lacking. The simulated results depend on a particular choice of kinetic parameters, while not only network topology but also parameter values alter the system's features [13]–[16]. To understand the global properties of circadian clocks, it is necessary to search all plausible kinetic parameter sets that generate circadian oscillations and to characterize the oscillatory features over all of the parameter sets. A shortage of the parameter search may lead to a wrong conclusion. To efficiently search parameters, we developed the two-phase search (TPS) method, which combines a random search with genetic algorithms to achieve global search while reducing computational cost [17].

Robustness is the ability to resume reliable operation in the face of different types of perturbations: parameter uncertainty, environmental and genetic changes, and stochastic fluctuations [18]–[20]. The importance for robustness is a functional criterion to characterize the performance of biochemical networks [21], and it can be used as a measure for determining plausibility among different competing models, assuming that biological designs enhance robustness. We proposed quasi-multiparameter sensitivity (QMPS) as a numerical and fast measure of robustness to the uncertainty of all kinetic parameters [22].

In general, feedback loops can be distinguished in terms of topological features: loop length, loop redundancy, and coupling types of multiple loops. It is known that a negative feedback with a long reaction chain generates an oscillator more readily than one with short chains [23]. By using TPS and QMPS, we demonstrated that long-chain feedback loop has potential to present a robust oscillator through the mechanism of distributed time delays [22]. In engineering and biology, redundancy is the main pillar of system's robustness. Genetic redundancy enables reliable development against fluctuating environment and mutations [24]–[27]. Redundant metabolic pathway reduces the sensitivity to enzyme activity for the flux and concentration of end products [22], [28]. In circadian clocks, it is critically important to understand how multiple, complex feedback loops are designed for robust oscillations. Stelling et al. characterized the robustness of three types of feedback models: the single, dual, and redundant feedback models by using multi-parameter perturbation analysis, suggesting that the most robust model is not the dual feedback model (employed by real biological systems) but the redundant feedback model (a hypothetical model) [15]. It may be a widely-recognized hypothesis, but a question arises: how the dual feedback architecture survives against the redundant feedback architecture despite less robustness of it.

On the other hand, the entrainment to a fluctuating environment was numerically analyzed to validate mathematical models for circadian clocks [5], [6], [8], [10]. Gonze and Goldbeter investigated the occurrence of various modes of dynamic behaviors as a function of the forcing period and of the amplitude with respect to light-induced changes in kinetic parameters [29]. Kurosawa and Goldbeter examined how the entrainment of these rhythms is affected by the free-running period (period under constant darkness) and by the amplitude of the external light-dark cycle [30]. However, their analyses largely depended on a particular choice of kinetic parameters. Considering that kinetic parameters constantly fluctuate within a cell, the network structure should be the main source of the capability of entrainment. Therefore, global, firm analysis is required to reveal how a particular structure in circadian clocks is related to light entrainment.

By using rigorous numerical methods of TPS and QMPS, we reveal the mechanism of how particular feedback architecture is related to robustness and to entrainment to light-dark cycles in circadian clocks. Furthermore, we demonstrate how the dual feedback architecture evolves out of many competing feedback systems, correcting or overcoming the existing hypothesis.

## Results and Discussion

### Biochemical Models

**Figure 1:**
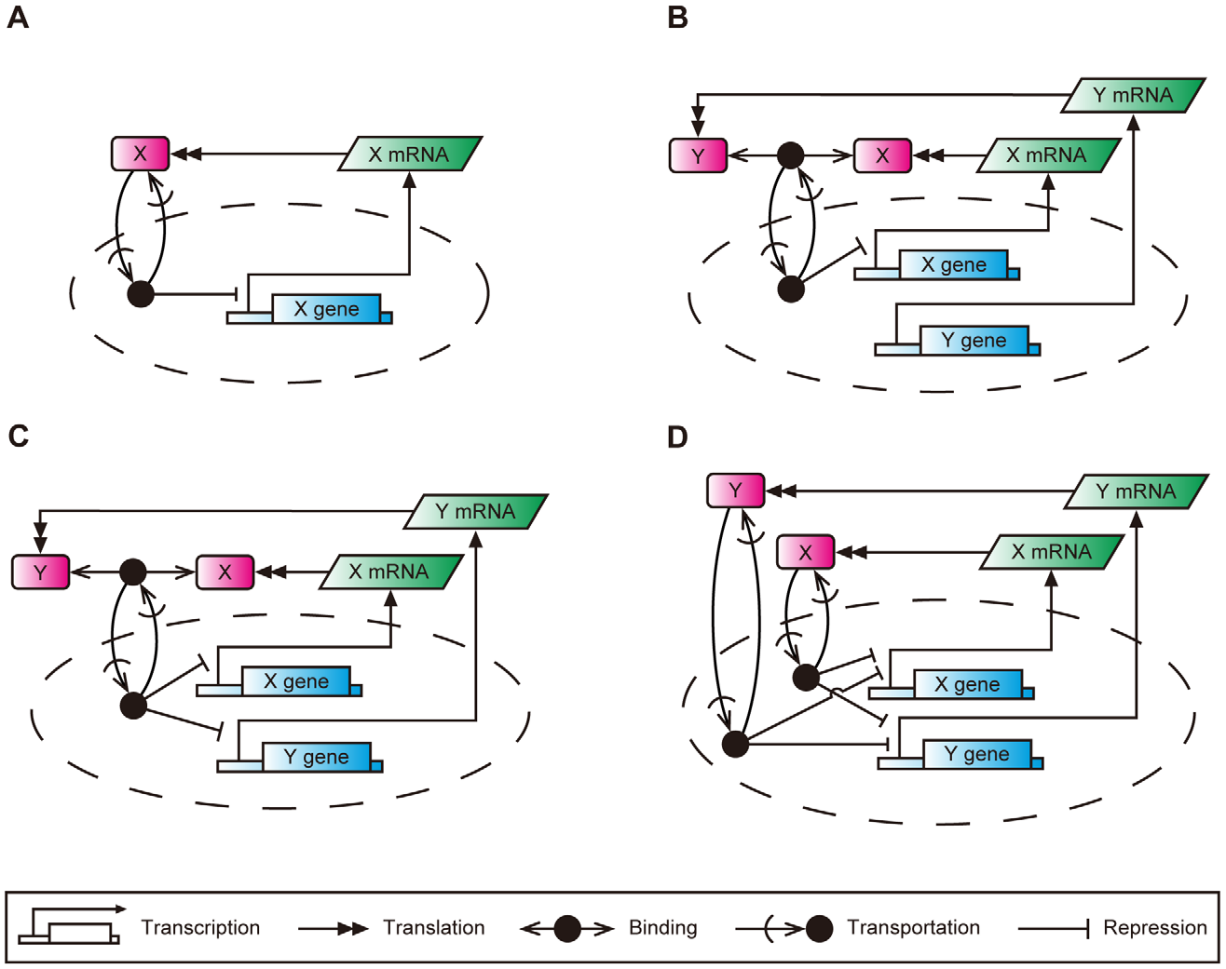
Biochemical network maps of the circadian clock models with different types of loop coupling logics. A: The single feedback model, B: the semi-dual feedback model, C: the dual feedback model, D: the redundant feedback model. The notation of CADLIVE [46]–[48] is used for simplifying the diagram. The dashed circle represents nucleus.

In many biological models feedback loops are connected in different manners. Architectures of feedback loops are featured by loop redundancy and coupling of multiple loops. To analyze how the loop coupling logic affects robustness and entrainment, the single, semi-dual, dual, and redundant feedback models were constructed by simplifying or refining the feedback models described elsewhere [4], [5], [12], [15]. The network maps are shown in Figure 1. The mathematical equations and their associated parameters are shown in Tables 1 and 2, respectively.

**Table 1:** Dynamic models of competing circadian clock models.

Model name	Equation	
The single feedback model (*m* = 3)	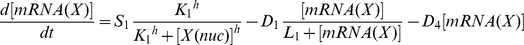	1
	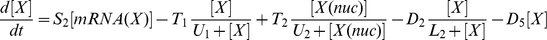	2
	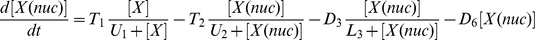	3
The semi-dual feedback model (*m* = 6)	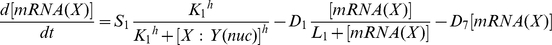	1
	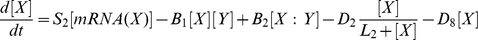	2
	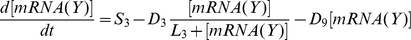	3
	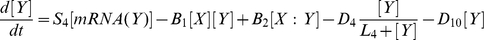	4
	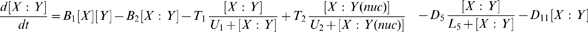	5
	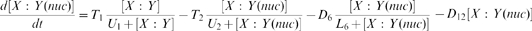	6
The dual feedback model (*m* = 6)	The equations except No. 3 are the same as those of the semi-dual feedback model.	
	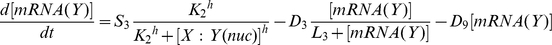	3
The redundant feedback model (*m* = 6)	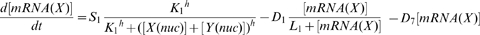	1
	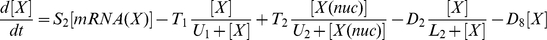	2
	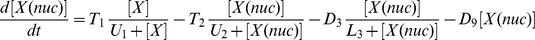	3
	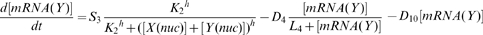	4
	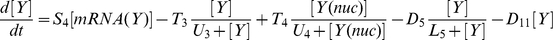	5
	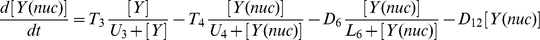	6

[*mRNA*(*X*)] is mRNA for protein *X*, [*X*] protein *X*, and [*X*(*nuc*)] protein *X* in nucleus. [*mRNA*(*Y*)], [*Y*], and [*Y*(*nuc*)] are named in the same manner. [*X*:*Y*] is the binding complex of *X* and *Y*, [*X*:*Y*(*nuc*)] the complex in nucleus. *m* is the number of equations for each model.

**Table 2:** List of kinetic parameters for competing circadian clock models.

Parameter	Definition	Value
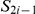 ( 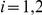 )	Maximum rates for transcriptions	1.0 [nM h^−1^]
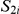 ( 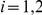 )	Translation rate constants	1.0 [h^−1^]
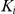 ( 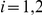 )	Affinity constants for transcriptions	1.0 [nM]
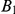	Association constant	1.0 [nM^−1^ h^−1^]
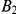	Dissociation constant	1.0 [h^−1^]
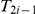 ( 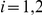 )	Maximum rates for transportations (cytoplasm to nucleus)	1.0 [nM h^−1^]
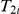 ( 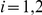 )	Maximum rates for transportations (nucleus to cytoplasm)	1.0 [nM h^−1^]
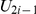 ( 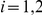 )	Affinity constants for transportations (cytoplasm to nucleus)	1.0 [nM]
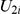 ( 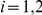 )	Affinity constants for transportations (nucleus to cytoplasm)	1.0 [nM]
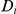 ( 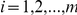 )	Maximum rates for degradations	1.0 [nM h^−1^]
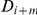 ( 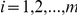 )	Degradation rate constants	0.01 [h^−1^]
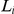 ( 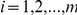 )	Affinity constants for degradations	1.0 [nM]
*h*	Hill coefficient	4.0 (fixed)

*m* is the number of equations for each model. Since the values of kinetic parameters are hardly measured in circadian oscillators, the followings are employed as the reference values.

The single feedback model (Figure 1A) is a simplified version of *Drosophila* PER feedback model [4]. In this model, *X* gene expression is negatively controlled by clock protein *X*. The single feedback model is used as the reference or control. The clock protein in nucleus is regarded as the output component from the circadian system as the clock proteins control gene expressions *in vivo*. In the single feedback model, *X* in nucleus (*X*(*nuc*)) is considered as the output component.

The semi-dual feedback model (Figure 1B) is a refined version of the dCLK-CYC feedback loop in the interlocked feedback model for *Drosophila* circadian clock [12]. In the semi-dual feedback model, the synthesis of protein *X* is negatively regulated by the heterodimer of *X*:*Y*, while the synthesis of protein *Y* occurs constitutively. In this model, the *X*:*Y* complex in nucleus (*X*:*Y*(*nuc*)) is considered as the output component. In *Drosophila*, *X* and *Y* correspond to dCLK and CYC, respectively. CYC is reported to be abundant compared to dCLK [31]. The question arises as to whether *Y* level in the semi-dual feedback model contributes to the performance in robustness and entrainment.

The dual feedback model (Figure 1C) is a simplified version of the *Drosophila* PER-TIM feedback model [5]. In this model, the *X* and *Y* feedback loops are coupled via the complex of *X*:*Y* and the syntheses of both *X* and *Y* are negatively regulated by *X*:*Y*. In *Drosophila*, *X* and *Y* correspond to PER and TIM. Unlike the semi-dual feedback model, the *Y* loop in the dual feedback model is closed to form a negative feedback control, where the *Y* concentration oscillates with *X*. In this model, the *X*:*Y* complex in nucleus (*X*:*Y*(*nuc*)) is considered as the output component. The dual feedback model has the symmetric structure of *X* and *Y* loops. By assigning the same values to the corresponding kinetic parameters of both the *X* and *Y* loops (e.g., *S*_1_ to *S*_3_, *D*_1_ to *D*_3_), the dual feedback model shows a kinetic symmetry between the *X* and *Y* loops.

The redundant feedback model (Figure 1D), which is not seen in organisms, was presented as a competitive model to the dual feedback model [15]. It has a symmetric structure between the *X* and *Y* loops, where *X* and *Y* independently regulate the syntheses of themselves. The total amount of *X* and *Y* in nucleus (*X*(*nuc*)+*Y*(*nuc*)) is the output. The redundant feedback model was mentioned to be the most robust oscillator for all the models.

### Robustness Analysis

We investigated the mechanism by which different coupling logics: the single, semi-dual, dual, and redundant feedback models provide robustness to perturbation to an entire system. To extract the global properties of those models, all plausible kinetic parameter sets that generate circadian oscillations in constant darkness are searched to characterize their oscillatory features. It is important that the conclusion is independent of a particular choice of kinetic parameter values. To this end, we used two-phase search (TPS) method and quasi-multiparameter sensitivity (QMPS). For details, see Materials and Methods.

### The semi-dual feedback model

**Figure 2:**
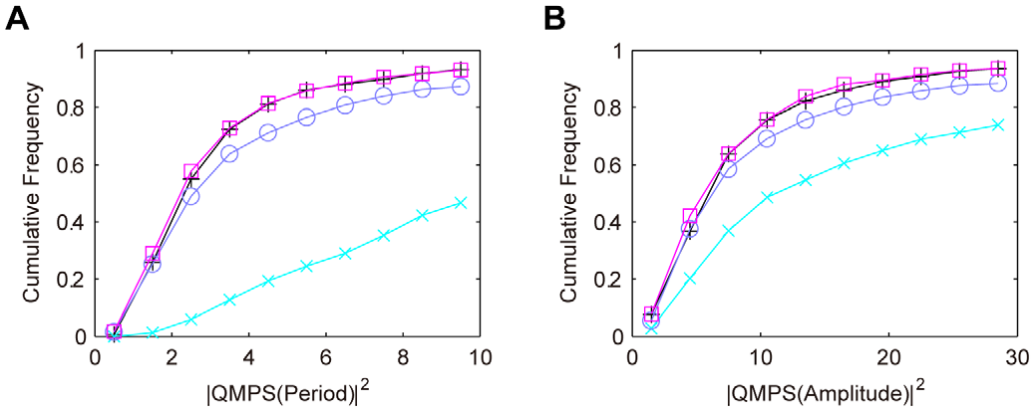
Cumulative frequency distributions of QMPS for the oscillatory behaviors in the semi-dual feedback model. A: QMPS for period, B: QMPS for amplitude. The level of protein *Y* was changed in the semi-dual feedback model: *Y*<10 nM (cross), 10 nM≤*Y*≤200 nM (circle), *Y*>200 nM (square). The single feedback model (plus) is the control model.

For the semi-dual feedback model, we simulated the QMPS of the period and amplitude of the oscillatory behaviors of the output component (*X*:*Y*(*nuc*)). Especially, we analyzed how the robustness depends on the *Y* level. The kinetic parameter values were searched by TPS so as to provide typical oscillatory behaviors: The period and amplitude for the oscillation of the output component were set to 23–25 h and to 2–6 nM, respectively. In the parameter search, the total *Y* level was constrained within the specific range (less than 10 nM, between 10 and 200 nM, or more than 200 nM). The search parameter values were varied by 0.1–10 fold with respect to the reference values as shown in Table 2. For each range of the *Y* level, a thousand of the solution parameter sets were obtained that produce the given oscillatory features. For all the solutions, QMPSs were calculated with respect to the period and amplitude of the output component oscillations. The cumulative frequency distributions for QMPS are shown in Figure 2. The QMPSs for period and amplitude decrease with an increase in the amount of *Y*, and their QMPS distributions approach to those in the single feedback model. With respect to robustness in oscillations, the semi-dual feedback model can be comparable to the single feedback model, but cannot overwhelm it.

The semi-dual feedback model was derived from the dCLK-CYC feedback loop in *Drosophila* [12], where *X* and *Y* correspond to dCLK and CYC, respectively. Our results predict that a high amount of CYC leads to robust oscillations. Indeed, the concentration of CYC is much higher than that of dCLK in *Drosophila* [31]. In mammals, BMAL1 and CLK appear to constitute a semi-dual feedback architecture [6], where *X* and *Y* correspond to BMAL1 and CLK, respectively. The level of CLK is higher than that of BMAL1 [32], [33]. These observations indicate that living systems have evolved to increase in the level of protein *Y* in order to cope with parameter uncertainty.

### The dual feedback model

The dual feedback model has the symmetric structure of the *X* and *Y* loops. Here parameter *ρ* (≥0) is introduced, which adjusts the symmetry between *X* and *Y* loops in terms of kinetics. In TPS, the search space for the kinetic parameters associated with the *Y* loop (*S_i_*_+2_, *i* = 1,2; *K*_2_; *D_i_*_+2_, *i* = 1,2,7,8; *L_i_*_+2_, *i* = 1,2) is provided by *ρ* and by the kinetic parameters associated with the *X* loop:
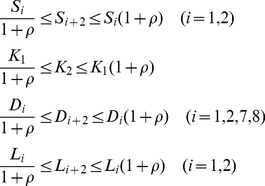
(1)As the value of *ρ* decreases, the values of the kinetic parameters for the *Y* loop approach to those for the *X* loop, increasing kinetic symmetry. When the value of *ρ* is zero, the values of the kinetic parameters associated with the *X* and *Y* loops are exactly the same and the two loops are perfectly symmetric. When the value of *ρ* is large enough (≥99), the kinetic parameter values for both the *X* and *Y* loops are independently assigned.

**Figure 3:**
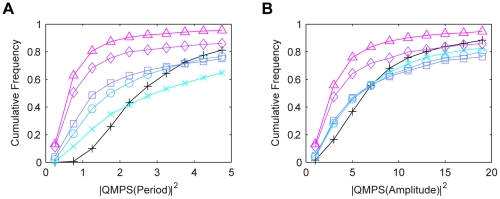
Cumulative frequency distributions of QMPS for the oscillatory behaviors in the dual feedback models. A: QMPS for period, B: QMPS for amplitude. The kinetic symmetry (*ρ*) was changed in the dual feedback model: *ρ*≥99 (cross), *ρ* = 1 (circle), *ρ* = 0.1 (square), *ρ* = 0.01 (diamond), *ρ* = 0 (triangle). The single feedback model (plus) is the control model. A decrease in *ρ* increases the kinetic symmetry.

We simulated the QMPS of the period and amplitude of the output component (*X*:*Y*(*nuc*)) of the dual feedback model with different values of *ρ*. The kinetic parameter values were searched by TPS so as to provide typical oscillatory behaviors, where they were varied by 0.1–10 fold with respect to the reference values as shown in Table 2. In addition, the search space for the kinetic parameters associated with the *Y* loop is further constrained by Eqs. (1). For each *ρ* value, a thousand of the solution parameter sets were obtained that produce the target oscillatory behaviors. For all the solutions QMPSs were calculated with respect to the period and amplitude of the output component oscillations. The cumulative frequency distributions for QMPS are shown in Figure 3. A decrease in the *ρ* value decreases the QMPS values with respect to both period and amplitude, indicating that the kinetic symmetry of feedback loops enhances the robustness of the oscillation to uncertainty of multiple parameters. When *ρ* equals to zero, the dual feedback model provides the most robust oscillator and greatly overwhelms the single feedback model.

Here, we present the hypothesis that the dual feedback model evolves toward the increased kinetic symmetry between the *X* and *Y* loops. A dual feedback architecture is found as the PER-TIM system in the *Drosophila*, and the symmetry between the two feedback loops are frequently assumed [5], [10], [11]. Although it is unclear whether these feedback loops are kinetically symmetric *in vivo*, many experimental data suggested that the processes of PER and TIM have the similar values of kinetic parameters. The E-box motif, which is a target for transcription factors dCLK and CYC, is located upstream of both the *per* and *tim* genes [34]–[37]. Therefore, the dCLK:CYC complex seems to have almost the same affinities to the *per* and *tim* promoters. The *per* and *tim* transcripts cycle in abundance with similar amplitudes and phases [38]. The time courses of PER and TIM are similar in shape and largely overlap [31], [39]. These experimental data support the hypothesis that the PER-TIM dual feedback system is designed to hold the kinetic symmetry, providing the robustness to uncertainty of kinetic parameters.

### The redundant feedback model

**Figure 4:**
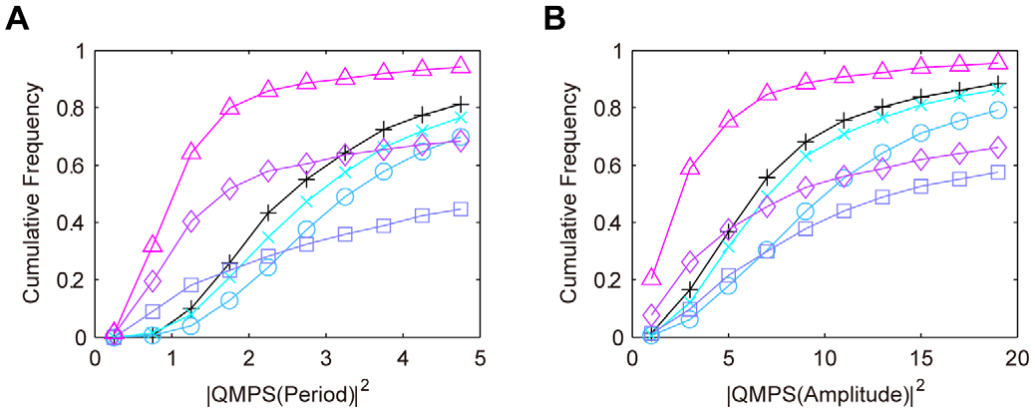
Cumulative frequency distributions of QMPS for the oscillatory behaviors in the redundant feedback models. A: QMPS for period, B: QMPS for amplitude. The kinetic symmetry (*ρ*) was changed in the redundant feedback model: *ρ*≥99 (cross), *ρ* = 1 (circle), *ρ* = 0.1 (square), *ρ* = 0.01 (diamond), *ρ* = 0 (triangle). The single feedback model (plus) is the control model. A decrease in *ρ* increases the kinetic symmetry.

The redundant feedback model has the symmetric structure of the *X* and *Y* feedback loops. As shown in the section for the dual feedback model, we use parameter *ρ* (≥0). In TPS, the search space for the kinetic parameters associated with the *Y* loop (*S_i_*_+2_, *i* = 1,2; *K*_2_; *T_i_*_+2_, *i* = 1,2; *U_i_*_+2_, *i* = 1,2; *D_i_*_+3_, *i* = 1,2,3,7,8,9; *L_i_*_+3_, *i* = 1,2,3) is determined by *ρ* and by the kinetic parameters associated with the *X* loop:
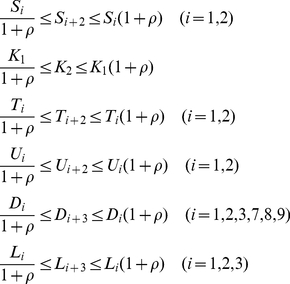
(2)The QMPS for the period and amplitude of the output (*X*(*nuc*)+*Y*(*nuc*)) was analyzed. The kinetic parameters were searched by TPS so as to provide typical oscillatory behaviors, where they were varied by 0.1–10 fold with respect to the reference values as shown in Table 2. In addition, the search space for the kinetic parameters associated with the *Y* loop is further constrained by Eqs. (2). For each *ρ* value, a thousand of the solution parameter sets were obtained. For all the solutions QMPSs were calculated with respect to the period and amplitude of the output oscillations. The cumulative frequency distributions for QMPS are shown in Figure 4. At a *ρ* value of more than 0.1, the QMPS values increase with a decrease in the *ρ* value. At a *ρ* value of smaller than 0.1, the QMPS values decrease with a decrease in *ρ*.

**Figure 5:**
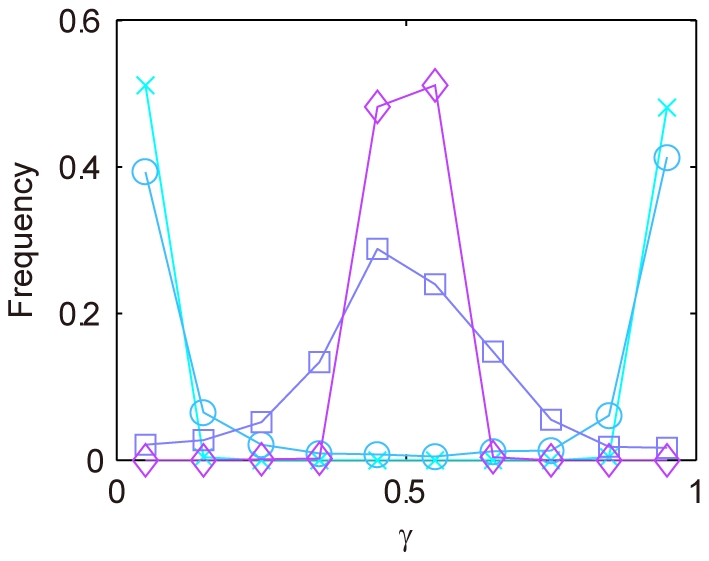
Frequency distributions of the *γ* values for the parameter sets that yield circadian oscillation. The frequency distributions of the quantitative balance between *X* and *Y* loops (*γ*) were simulated, while changing the kinetic symmetry (*ρ*): *ρ*≥99 (cross), *ρ* = 1 (circle), *ρ* = 0.1 (square), *ρ* = 0.01 (diamond). A decrease in *ρ* increases the kinetic symmetry. *γ* is the quantitative balance of the *X* and *Y* feedback loops, which is defined by: 
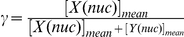
, where [*X*(*nuc*)]*_mean_* indicates the mean concentration for *X* in nucleus and [*Y*(*nuc*)]*_mean_* that for *Y* in nucleus. When *γ* is close to zero, the effect of the *X* loop on the oscillator is weak, while the *Y* loop is dominant. When *γ* is close to 0.5, the effects of both the *X* and *Y* loops are comparable. When *γ* is close to one, the *X* loop is dominant. At *ρ* = 0 (perfect kinetic symmetry), *γ* is always equal to 0.5. The distribution for *ρ* = 0 is not shown.

To understand the complex behaviors shown in Figure 4, the quantitative balance of the *X* and *Y* feedback loops *γ* is defined by:
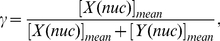
(3)where [*X*(*nuc*)]*_mean_* indicates the mean concentration for *X* in nucleus and [*Y*(*nuc*)]*_mean_* that for *Y* in nucleus. When *γ* is close to zero, the effect of the *X* loop on the oscillator is weak, while the *Y* loop is dominant. When *γ* is close to 0.5, the effects of both the *X* and *Y* loops are comparable. When *γ* is close to one, the *X* loop is dominant. The *γ* distributions for the redundant feedback model with respect to *ρ* are shown in Figure 5. When *ρ* is set to a large value, the values of *γ* are biased towards zero or one, indicating that kinetic symmetry is not generated: the level of *X* in nucleus oscillates with a negligible level of *Y* in nucleus, and vice versa. A value of *γ* approaches to 0.5 with a decrease in *ρ*. A *ρ* value of less than 0.01, strong confinement to kinetic symmetry, is required to generate the oscillations with the comparable levels of *X* and *Y* in nucleus.

As shown in Figure 4, the distribution of QMPS for the redundant feedback model is the same as that of the single feedback model at a large value of *ρ*, where *γ* is almost zero or one (Figure 5). Such an asymmetric redundant feedback model can be the same as the single feedback model. Under a weak constrain to kinetic symmetry or a *ρ* value of more than 0.1, QMPS increases with a decrease in *ρ*. It is probably because the oscillatory behaviors of the *X* and *Y* loops would interfere with each other, i.e., the intrinsic cycles of the two loops are not consistent. Under the strong constraint to kinetic symmetry or a *ρ* value of smaller than 0.1, the QMPS values decrease with a decrease in *ρ*. When *ρ* is set to zero, the frequency distribution is biased toward a small value of QMPS. The redundant feedback model with exact kinetic symmetry became a more robust oscillator to uncertainty of all kinetic parameters than the single feedback model. The redundant feedback model provides robustness, when the kinetics of both the feedback loops is symmetric or either of the two feedback loops is negligible or dominant (a virtual single feedback model). Under the other conditions, the robustness is readily decreased, probably because the cycles by the two feedback loops interfere with each other.

The redundant feedback model has the potential to produce more robust oscillation than that of the single feedback model. However, the redundant feedback model seems to have difficulty in evolving toward the kinetic symmetry of the two feedback loops, because a break in the symmetry readily destroys the robust oscillations (e.g. *ρ* = 0.1). This may be the reason that redundant feedback oscillators are not seen in biology.

In the dual and redundant feedback models, the two feedback loops should coordinate to oscillate. Otherwise, the cycles of both the feedback loops interfere with each other. Kinetic symmetry is thus required for robust oscillators. In the dual feedback model, the two feedback loops are tightly coordinated by binding the two proteins. In the redundant feedback model, the two feedback loops are loosely connected at the transcription regulations. Different types of loop coupling alter the robustness.

### Comparison among the feedback models

**Figure 6:**
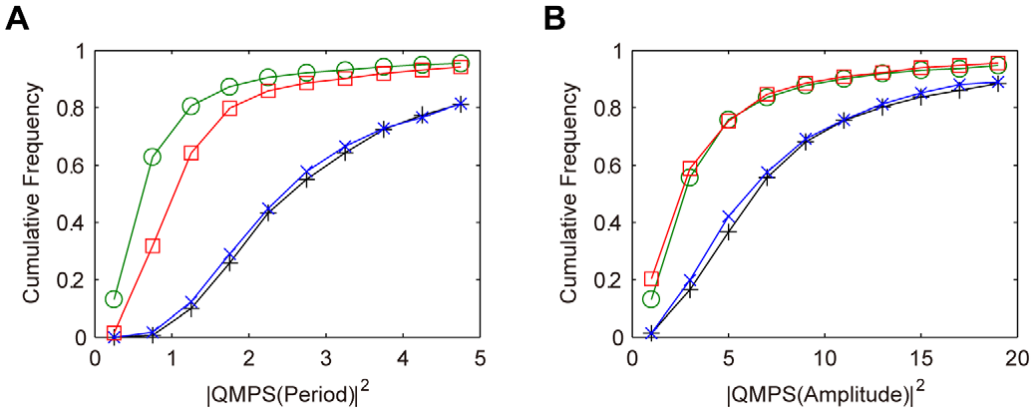
Cumulative frequency distributions of QMPS for the oscillatory behaviors in the competing models. A: QMPS for period, B: QMPS for amplitude. The single feedback model (plus), the semi-dual feedback model with *Y*>200 nM (cross), the dual feedback model with *ρ* = 0 (circle), the redundant feedback model with *ρ* = 0 (square). *ρ* = 0 indicates perfect symmetry between two feedback loops.

In summary, we compared the QMPS distributions for the single, semi-dual, dual, and redundant feedback models. The QMPS distributions with respect to period and amplitude are shown in Figure 6. The distributions for the single and semi-dual feedback models are almost identical. When the *ρ* value is set to zero, the QMPS values of amplitude for the dual and redundant feedback models are comparable and lower than other models. With respect to period, the QMPS values of the dual feedback models are lower than any other model. The dual feedback model can be the most robust oscillator.

In [15], the authors used the Monte Carlo simulations to compare the robustness to uncertainty of all parameters between the competing models: the PER single feedback model [4], the PER-TIM dual feedback model [5], and the PER-TIM redundant feedback model. They suggested that the redundant feedback model is the most robust oscillator for these models and the dual feedback model is less robust than the single feedback model. However, our extensive analysis presents an alternative hypothesis to the existing one: the dual feedback model has the potential to provide the most robust oscillator to multiple parameter perturbations. The parameter search in their work [15] was very short, where they used only dozens of parameter sets with respect to each model. In the Monte Carlo method, they run only 200 simulations for each parameter set. To reliably compare alternative models, the efficient search for large parameter space is required and at least ten thousand simulation runs are needed for each reference parameter set [22].

### Entrainment Probability Maps for the Feedback Models

Since the values of kinetic parameters constantly fluctuate within a cell, the entrainment of circadian rhythms should not rely on the fine-tuned values of them. To elucidate a mechanism by which oscillations are entrained to light-dark (LD) cycles, we performed the entrainment analysis [29] for all the kinetic parameter sets used in the section of Robustness Analysis. In *Drosophila*, light induces the degradation of clock protein TIM, which allows circadian oscillations to entrain to a diurnal cycle [40]–[42]. To consider the light-increased degradation rate for the clock protein, the maximum rate for degradation of *X* was increased during light phase. The increase rate in the parameter and the forcing period of LD cycles are denoted as *δ* and *ζ*, respectively. For each parameter set that produces typical circadian rhythm in constant darkness, the region where oscillations are successfully entrained is provided as a function of *ζ* and *δ*. The probability of entrainment at point *ζ*-*δ* is determined by counting the number of parameter sets that successfully entrain to LD cycles with *ζ* and *δ*. For details, see Materials and Methods.

**Figure 7:**
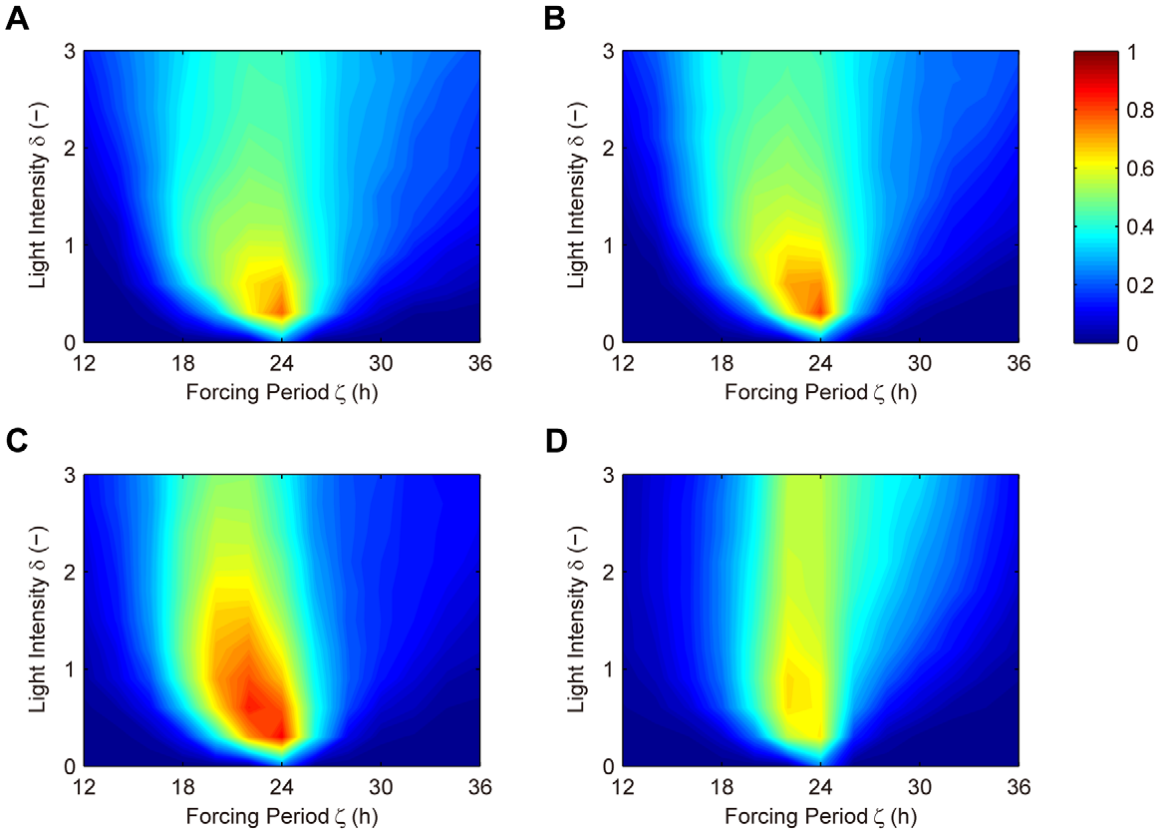
Entrainment probability maps for different types of feedback models. A: the single feedback model, B: the semi-dual feedback model with *Y*>200 nM, C: the dual feedback model with *ρ* = 0, D: the redundant feedback model with *ρ* = 0. *ρ* = 0 indicates perfect symmetry between the two feedback loops. The color indicates the probability of entrainment given by Eq. (9), the ratio of the parameter sets that entrain to light-dark cycles to all the parameter sets. Red color indicates a high probability, where the cycle readily entrains to the light-dark cycle; blue a low one.

For the single, semi-dual, dual, and redundant feedback models, the entrainment probability map was drawn with respect to the period of LD cycles (*ζ*) and the intensity of light (*δ*). The entrainment probability map for the single feedback model is shown in Figure 7A. The single feedback model entrains 24 h LD cycle, which is almost equal to the free-running period (the period under constant darkness). When the period of LD cycles is far from 24 h, irregular oscillations (quasi-periodic and chaotic oscillations) occur and cannot entrain to LD cycles. Entrainment occurs with relatively weak light stimuli, while strong light causes irregular oscillations. The maps for the semi-dual feedback model with various concentrations of *Y* are shown in Figure S1. The change in the level of *Y* does not dramatically affect ability of entrainment. At a *Y* level of more than 200 nM (Figure 7B) the entrainment probability map is similar to that of the single feedback model. In the dual feedback model, at *ρ* of more than one, kinetic symmetry increases the probability of entrainment (Figure S2). The kinetically symmetric dual feedback model (*ρ* = 0) (Figure 7C) readily entrains to LD cycles in the widest space of the forcing period and light intensity. The dual feedback model with kinetic symmetry is the most reasonable choice for light entrainment. In the redundant feedback model, kinetic symmetry increases the probability of entrainment (Figure S3), but the perfect kinetic symmetry (*ρ* = 0) (Figure 7D) does not entrain to LD cycles more than the single feedback model.

For all the models, forcing periods away from the free-running period or strong light stimuli caused irregular oscillations. These results are consistent with the previous works [29], [30]. It is important for successful entrainment to incorporate the forcing period in intrinsic oscillations while avoiding irregular oscillations. In the dual feedback model, the light-perturbed *X* loop and the unperturbed *Y* loop are merged at the complex *X*:*Y* that regulates the transcriptions of *X* and *Y*. This coupling mechanism is suggested to effectively incorporate the forcing period into the system, avoiding irregular oscillations. In the redundant feedback model, since the *X* and *Y* loops separately regulate transcriptions, the difference in the oscillations between the light-perturbed *X* loop and the unperturbed *Y* loop is directly transmitted to the transcription regulations, thereby causing irregular oscillations or failure in the entrainment to LD cycles.

### Key Devices for Robust and Entrainable Oscillators

The complex feedback system of the circadian clocks is one of the most extensively studied systems, which generates the robustness to the uncertainty of kinetic parameters and entrains to periodic changes in the environment. However, it remains unclear how such complex feedback loops are designed, while the single or simple feedback can be a sufficiently robust and entrainable oscillator. By performing global numerical analyses by TPS, we demonstrated that the dual feedback model is the most reasonable choice for creating a robust and entrainable oscillator out of various types of loop couplings, which corrects or overcomes the existing hypothesis [15]. The dual feedback model, employed by a real biological system, provides the most robust oscillator and readily entrains LD cycles. Furthermore kinetic symmetry in the dual feedback model enhances the robustness with respect to uncertainty of multiple parameters and the ability to entrain to a LD cycle, providing an insight on the mechanism by which the dual feedback model evolves toward kinetic symmetry for enhanced robustness and entrainments. We discovered smart, intelligent devises hidden in the real biological circadian oscillators. The key devices are the coupling of two feedback loops by forming a protein complex and kinetic symmetry between them, which generate remarkable robustness to perturbations within a cell and enable entrainment to a wide range of light signals.

## Materials and Methods

### Mathematical Comparison between Competing Models

In order to understand the mechanism by which a specific regulation generates a particular cellular function, it is important to compare the performance criteria between the competing models: the model containing the specific regulation and that without it. While system's properties are affected by not only the network structure but also the values of the kinetic parameters, the conclusion of the comparison should be independent of a change in parameters [15], [22], [43]. In competing models, the kinetic parameter values are searched within a certain range by the two-phase search (TPS) method to find all possible solutions that generate target behaviors. TPS is presented to systematically analyze the dynamic behaviors in a large parameter space by searching all plausible parameter values without any biases [17]. TPS consists of a random search and an evolutionary search (genetic algorithms: GAs) to effectively explore all possible solution sets of kinetic parameters satisfying a target or desired dynamics. The algorithm of TPS is described in Text S1 and Figure S4.

Our mathematical comparison method is basically the same as mathematically controlled comparison introduced by Alves and Savageau [43], where parameter search is randomly performed to guarantee equivalence among alternative models, and then the models are statistically compared with respect to the property of interest. First, we search the values of the kinetic parameters providing circadian oscillations to hold behavioral equivalence among all feedback models. Second, we statistically compare the distributions of QMPS among the feedback models. Alves and Savageau employed random search to obtain the values of kinetic parameters yielding biologically plausible behaviors, allowing fare comparison among alternative models. Instead of random search, we use TPS to greatly reduce computational cost. The distributions of solutions obtained by TPS were demonstrated to be statistically the same as those by random search [17].

### Quasi-multiparameter Sensitivity (QMPS)

Generally a dynamic model for biochemical networks is formulated by ordinary differential equations:
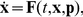
(4)where *t* is time, **x** is the vector whose elements are the variables for molecular concentrations, **p** = (*p*_1_,*p*_2_,…,*p_n_*) is the kinetic parameter vector, and *n* is the number of kinetic parameters. Let *q*(**p**) be a given target function. A single-parameter sensitivity of the target function with respect to a change in the *i*th parameter is defined as:
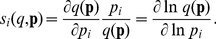
(5)A single-parameter sensitivity identifies sensitive or insensitive reactions to a target function in a biochemical network, while it yields only linear approximations of the target function to single parameter perturbation. Single-parameter sensitivity analysis does not estimate the robustness to the uncertainty of all parameters. Assuming that the relative change in the target function is linear to a change in each parameter, multiparameter sensitivity (MPS) [44], [45] is defined by:
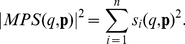
(6)For complex models (including Table 1), it is generally hard to analytically compute MPS. As a practical solution, quasi-multiparameter sensitivity (QMPS) is proposed, where the single-parameter sensitivities are numerically simulated by providing a small perturbation to a kinetic parameter. When each kinetic parameter *p_i_* is perturbed as given by 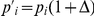, MPS is defined as the square sum of single-parameter sensitivities: 
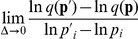
, where Δ approaches to zero. On the other hand, QMPS is defined by the square sum of 
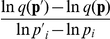
, where Δ is not nearly zero but a small value, e.g. Δ = 0.001. The algorithm for computing QMPS is described in Text S2. In this study the single-parameter sensitivities are calculated by setting the value of Δ to 0.001. Since the calculated values are consistent at Δ of less than 0.01, they can be used as the single-parameter sensitivities.

QMPS is not only computationally efficient but also consistent with the normalized variance for the target function obtained by Monte Carlo method. In general the Monte Carlo method is used to quantify robustness to multiple parameter perturbations, where all kinetic parameters are simultaneously varied and the normalized variance for the target function can be used as the indicator for robustness. On the other hand, QMPS is theoretically defined under the condition that a change in all parameter values is infinitesimal, while QMPS is demonstrated to practically be available under the condition that variations in all kinetic parameters are less than 10%. By using many mathematical models, we demonstrated that QMPS is consistent with the normalized variance by the Monte Carlo method even if the rate of variations in all kinetic parameters is set to as high as 10% [22].

QMPS is employed with TPS, thereby enabling the numerical comparison of robustness among alternative or competing models. First, we search the kinetic parameter values by TPS so as to provide typical oscillatory behaviors: in constant darkness the period and amplitude for the oscillation of the output component are set to 23–25 h and to 2–6 nM, respectively. The fitness function is described in Text S3. In general, oscillations with approximately 24 h period and large amplitude are regarded as typical circadian oscillation. The target period and amplitude are provided based on experimental data and theoretical studies [4], [5], [31], and their ranges are conveniently determined to obtain many solutions. Narrow ranges provide only a small number of solutions due to calculation complexity. Second, the QMPSs are calculated for all the solution parameter sets. When comparing the robustness of two alternative dynamic models, one model with a lower value of QMPS is more robust than the other. In this analysis the cumulative frequency is used as a function of the value of QMPS squared in order to characterize the robustness among alternative models with a variety of kinetic parameter values, generated by TPS. The cumulative frequency (*CF*) of a QMPS value *x* is given by:
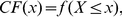
(7)where *X* is a random variable and *f* represents the frequency that *X* takes on a value less than or equal to *x*. According to this criterion, a higher cumulative frequency distribution indicates higher robustness and a higher cumulative frequency at a lower QMPS provides higher robustness. When two cumulative frequency curves to be compared intersect, statistical analysis by median is used instead of an intuitive analysis. The median corresponds to the value of QMPS that provides a cumulative frequency of 0.5. A low value of median provides high robustness.

### Entrainment Probability Map

To elucidate a mechanism of how oscillations are entrained to light-dark (LD) cycles, the entrainment analysis [29] is performed for all the kinetic parameter sets, searched by TPS, that generate typical oscillations in constant darkness. In *Drosophila*, light induces the degradation of clock protein TIM, which allows circadian oscillations to entrain to a diurnal cycle [40]–[42]. To consider the light-increased degradation rate for the clock protein, the maximum rate for degradation of clock protein *X* is increased during light phase as follows:
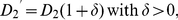
(8)where *δ* is the factor that increases *D*_2_. In addition to the degradation of *X*, in the semi-dual and dual feedback models, the degradation of the complex of *X* and *Y* (*X*:*Y*) is enhanced in the same manner as Eq. (8). The forcing period of LD cycles is defined as *ζ*. Light and dark phases in the LD cycle are set to the same in length. Using all the parameter sets that produce typical circadian rhythms in constant darkness, the region where oscillation is successfully entrained is provided as a function of *ζ* and *δ*. The probability of entrainment at point *ζ*-*δ* is determined by:
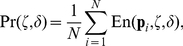
(9)where **p** *_i_* is the *i*th parameter set, *N* is the total number of the parameter sets, and En is the function that gives one when successful entrainment occurs, otherwise zero. By “successful entrainment”, we mean that period and amplitude of oscillations are constant (less than 1% and 10% of deviations, respectively) and the period of oscillations is the same as the forcing period (less than 1% of deviation).

## Supporting Information

Figure S1 **Entrainment probability maps for the semi-dual feedback model with various amounts of** ***Y*** **.** A: *Y*<10 nM, B: 10 nM≤*Y*≤200 nM, C: *Y*>200 nM. The color indicates the probability of entrainment given by Eq. (9), the ratio of the parameter sets that entrain to light-dark cycles to all the parameter sets. Red color indicates a high probability, where the cycle readily entrains to the light-dark cycle; blue a low one.
(PNG)

Figure S2 **Entrainment probability maps for the dual feedback model with various** ***ρ*** **values.** A: *ρ*≥99, B: *ρ* = 1, C: *ρ* = 0.1, D: *ρ* = 0.01. E: *ρ* = 0. A decrease in *ρ* increases the kinetic symmetry. The color indicates the probability of entrainment given by Eq. (9), the ratio of the parameter sets that entrain to light-dark cycles to all the parameter sets. Red color indicates a high probability, where the cycle readily entrains to the light-dark cycle; blue a low one.
(PNG)

Figure S3 **Entrainment probability maps for the redundant feedback model with various** ***ρ*** **values.** A: *ρ*≥99, B: *ρ* = 1, C: *ρ* = 0.1, D: *ρ* = 0.01. E: *ρ* = 0. A decrease in *ρ* increases the kinetic symmetry. The color indicates the probability of entrainment given by Eq. (9), the ratio of the parameter sets that entrain to light-dark cycles to all the parameter sets. Red color indicates a high probability, where the cycle readily entrains to the light-dark cycle; blue a low one.
(PNG)

Figure S4 **Initial population for genetic algorithm (GA) in the two-phase search (TPS) method.** In this figure, the dimension of parameter space is assumed to be two. **L** = (*L*_1_,*L*_2_) and **U** = (*U*_1_,*U*_2_) are the lower and upper bound vectors, respectively. RS stands for random search.
(PNG)

Text S1 **The algorithm of the two-phase search (TPS) method.**
(PDF)

Text S2 **The algorithm for computing quasi-multiparameter sensitivity (QMPS).**
(PDF)

Text S3 **The fitness function for circadian oscillations.**
(PDF)
