# Supplementary figures and images for: A Symmetric Dual Feedback System Provides a Robust and Entrainable Oscillator
Source: PLoS One. 2012 Feb 20;7(2):e30489. doi: 10.1371/journal.pone.0030489 (PMC3282687; doi:10.1371/journal.pone.0030489)

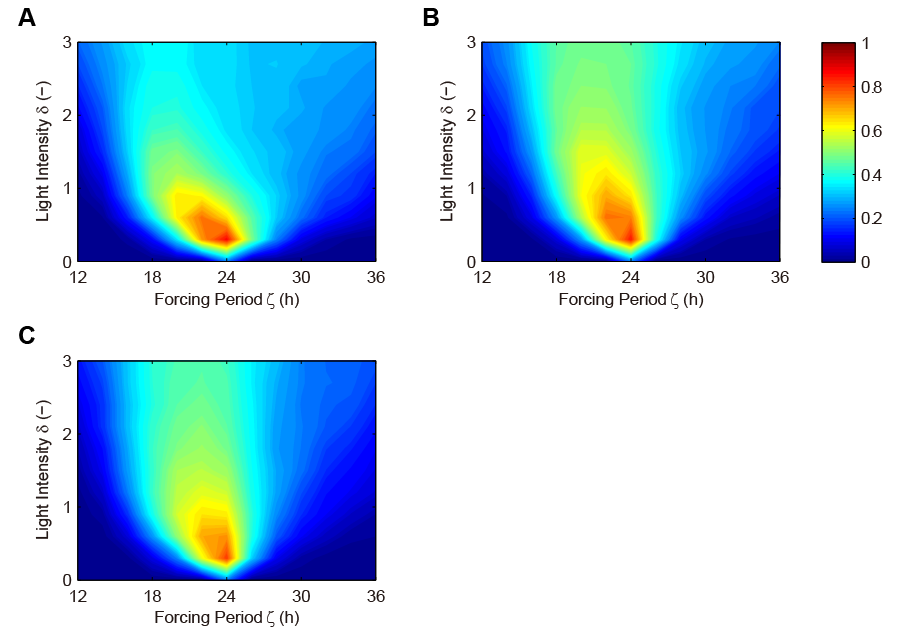

Supplement: Figure S1 — Entrainment probability maps for the semi-dual feedback model with various amounts of Y . A: Y<10 nM, B: 10 nM≤Y≤200 nM, C: Y>200 nM. The color indicates the probability of entrainment given by Eq. (9), the ratio of the parameter sets that entrain to light-dark cycles to all the parameter sets. Red color indicates a high probability, where the cycle readily entrains to the light-dark cycle; blue a low one. (PNG) [file pone.0030489.s001.png]

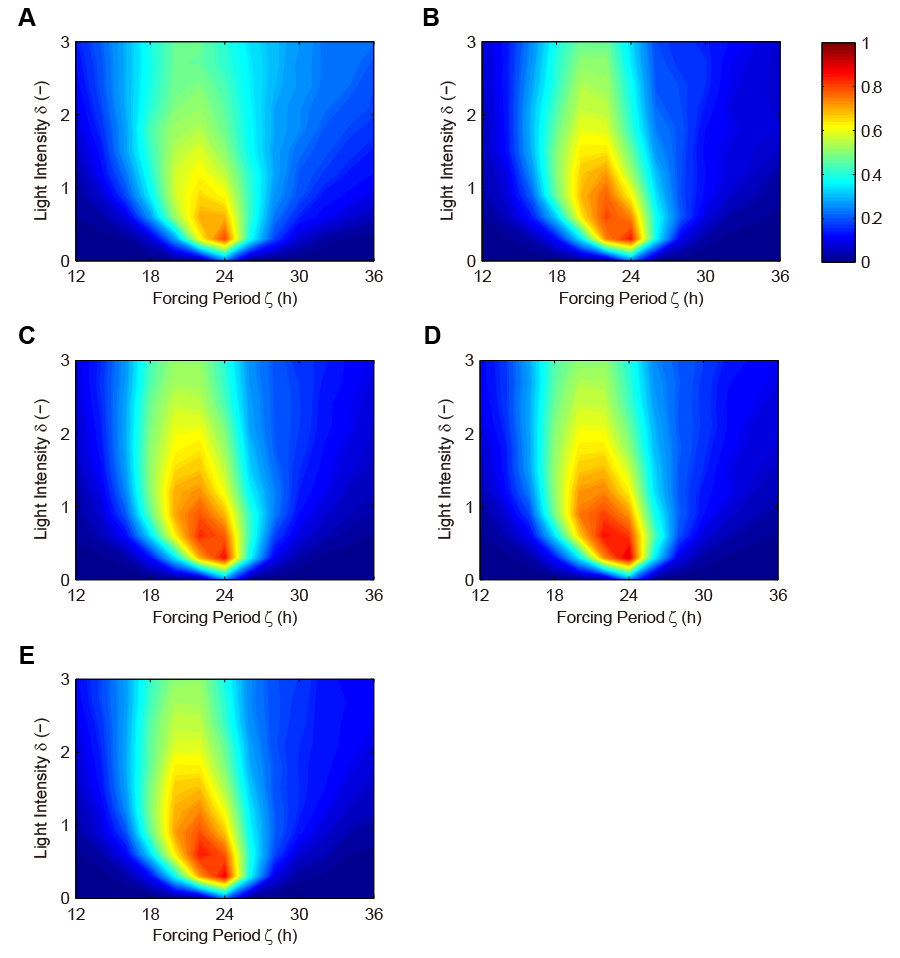

Supplement: Figure S2 — Entrainment probability maps for the dual feedback model with various ρ values. A: ρ≥99, B: ρ = 1, C: ρ = 0.1, D: ρ = 0.01. E: ρ = 0. A decrease in ρ increases the kinetic symmetry. The color indicates the probability of entrainment given by Eq. (9), the ratio of the parameter sets that entrain to light-dark cycles to all the parameter sets. Red color indicates a high probability, where the cycle readily entrains to the light-dark cycle; blue a low one. (PNG) [file pone.0030489.s002.png]

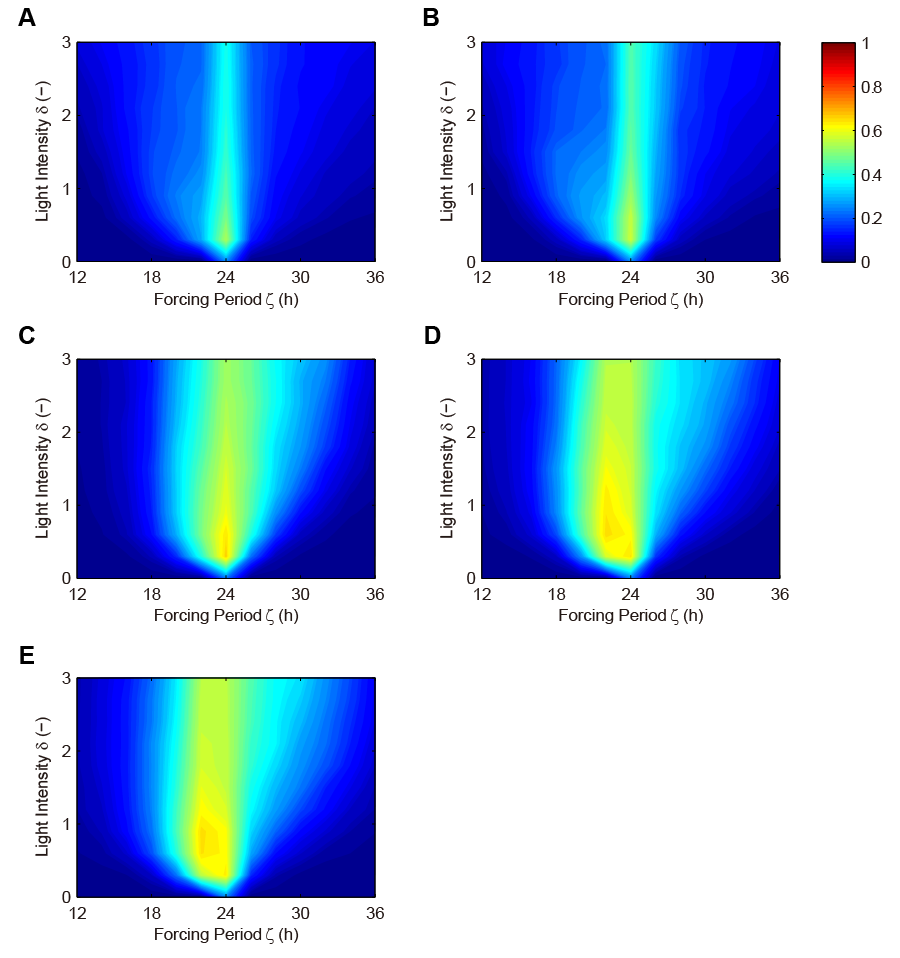

Supplement: Figure S3 — Entrainment probability maps for the redundant feedback model with various ρ values. A: ρ≥99, B: ρ = 1, C: ρ = 0.1, D: ρ = 0.01. E: ρ = 0. A decrease in ρ increases the kinetic symmetry. The color indicates the probability of entrainment given by Eq. (9), the ratio of the parameter sets that entrain to light-dark cycles to all the parameter sets. Red color indicates a high probability, where the cycle readily entrains to the light-dark cycle; blue a low one. (PNG) [file pone.0030489.s003.png]

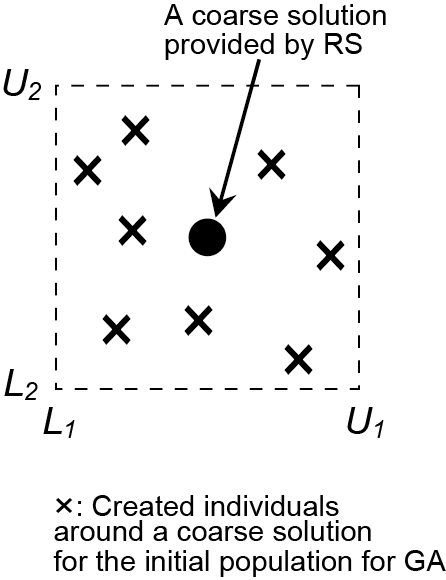

Supplement: Figure S4 — Initial population for genetic algorithm (GA) in the two-phase search (TPS) method. In this figure, the dimension of parameter space is assumed to be two. L = (L 1,L 2) and U = (U 1,U 2) are the lower and upper bound vectors, respectively. RS stands for random search. (PNG) [file pone.0030489.s004.png]
