# Supplementary material for: A Symmetric Dual Feedback System Provides a Robust and Entrainable Oscillator
Source: PLoS One. 2012 Feb 20;7(2):e30489. doi: 10.1371/journal.pone.0030489 (PMC3282687; doi:10.1371/journal.pone.0030489)
Supplement: Text S1 — The algorithm of the two-phase search (TPS) method. (PDF) [file pone.0030489.s005.pdf]

Text S1. The algorithm of the two-phase search (TPS) method

The two-phase search (TPS) method is presented to systematically search all plausible parameter vectors satisfying a target or desired property without any biases in a mathematical model (Maeda and Kurata, IPSJ Trans Bioinfo, 2009). TPS consists of a random search (RS) and genetic algorithms (GAs). First, the RS explores a large parameter space without any biases to find a coarse solution showing a good fitness value. The resultant coarse solution is employed to generate the initial populations for the subsequent GAs. Second, after the initial population is created around the coarse solution vectors, GAs intensively search all plausible solutions. Let $\mathbf{p} = (p_1, p_2, \ldots, p_n)$ be the parameter vector and Fitness(**p**) be the fitness function that evaluates how well the parameters reproduce the experimental behaviors. A low value of Fitness(**p**) means high consistency between the simulated time course and experimental data. The end condition of the first phase is provided by:

$$\text{Fitness}(\mathbf{p}) \le AEC\,, \quad \cdots \quad \text{(S1)}$$

where AEC is the allowable error for the coarse solutions. The initial population for the GA is randomly generated around a coarse solution (**Figure S4**). The lower and upper bound vectors for the initial population are derived from the coarse solutions as follows:

$$\mathbf{L} = (L_1, L_2, \ldots, L_n) \text{ and } \mathbf{U} = (U_1, U_2, \ldots, U_n)\,, \quad \cdots \quad \text{(S2)}$$

where L_i and U_i are determined by using a coarse solution $\mathbf{p}^c = (p_1^c, p_2^c, \ldots, p_n^c)$:

$$\begin{aligned} L_i &= p_i^c \cdot r^{-1} \\ U_i &= p_i^c \cdot r \end{aligned}\,, \quad \cdots \quad \text{(S3)}$$

where r is the factor that expands the region. The end condition of the second phase for a final solution is given by:

$$\text{Fitness}(\mathbf{p}) \le AE\,, \quad \cdots \quad \text{(S4)}$$

where AE is the allowable error of plausible solution ($AE \le AEC$). In this study, AE and r are set to zero and $10^{0.5}$, respectively. The values of AEC are set to a value from 0.1 to 0.6, depending on models. We confirmed that the settings of AEC values do not affect our results in this study.

The pseudocode for TPS is shown in the below box, where n_p and n_g are the population size and the maximum generation for GAs, respectively.

```
N ← 0
While N < Required number of solutions:
    # First Phase
    Randomly generate parameter vector p
    If Fitness(p) ≤ AE:
        Store p as a solution
        N ← N + 1
    Else if Fitness(p) ≤ AEC:
        # Second Phase
        As an initial population, randomly create n_p − 1 individuals between L and U
        Add p to the initial population
        Generation ← 1
        While Generation ≤ n_g :
            If the minimum value of fitness in population ≤ AE:
                Store the individual that gives the minimum fitness as a solution
                N ← N + 1
                Break
            End
            Execute selection, crossover, and mutation
            Generation ← Generation + 1
        End
    End
End
```
