# Supplementary material for: A Symmetric Dual Feedback System Provides a Robust and Entrainable Oscillator
Source: PLoS One. 2012 Feb 20;7(2):e30489. doi: 10.1371/journal.pone.0030489 (PMC3282687; doi:10.1371/journal.pone.0030489)
Supplement: Text S2 — The algorithm for computing quasi-multiparameter sensitivity (QMPS). (PDF) [file pone.0030489.s006.pdf]

Text S2. The algorithm for computing quasi-multiparameter sensitivity (QMPS)

Let $\mathbf{p} = (p_1, p_2, \ldots, p_n)$ be a kinetic parameter vector and $\mathbf{p}' = (p_1', p_2', \ldots, p_n')$ be a perturbed parameter vector, where n is the number of kinetic parameters. $q(\mathbf{p})$ is the target function (i.e. period or amplitude). Δ is the perturbation values given to each parameter ($\Delta = 0.001$ in this study). The pseudocode for the calculation of QMPS is shown in the below box. The final value of *sum* is $\left|QMPS(q, \mathbf{p})\right|^2$.

Run simulation with $\mathbf{p}$ and get $q(\mathbf{p})$

$sum \leftarrow 0$

For i from 1 to n:

  $\mathbf{p}' \leftarrow \mathbf{p}$ # This line refreshes $\mathbf{p}'$

  $p_i' \leftarrow p_i(1+\Delta)$

  Run simulation with $\mathbf{p}'$ and get $q(\mathbf{p}')$

  $$sum \leftarrow sum + \left(\frac{\ln q(\mathbf{p}') - \ln q(\mathbf{p})}{\ln p_i' - \ln p_i} \right)^2$$

End
