# Supplementary material for: A Symmetric Dual Feedback System Provides a Robust and Entrainable Oscillator
Source: PLoS One. 2012 Feb 20;7(2):e30489. doi: 10.1371/journal.pone.0030489 (PMC3282687; doi:10.1371/journal.pone.0030489)
Supplement: Text S3 — The fitness function for circadian oscillations. (PDF) [file pone.0030489.s007.pdf]

Text S3. The fitness function for circadian oscillations

To obtain the kinetic parameters providing typical oscillatory behaviors, the fitness function evaluates four properties: the absolute amplitude, relative amplitude, period, and regularity of oscillations. The absolute amplitude is defined as the difference between the peak and trough of oscillations of an output component:

$$AbsAmplitude = peak - trough \,. \quad \text{(S5)}$$

The relative amplitude is defined as the ratio of the absolute amplitude to the mean concentration of the output component:

$$RelAmplitude = 2.0 \cdot \frac{peak - trough}{peak + trough} \,. \quad \text{(S6)}$$

Period is defined as the time difference between the neighboring peaks of oscillations. The regularity of oscillation means whether the oscillation is regular (constant period and constant amplitude) or irregular (quasi-periodic or chaotic). The fitness function is provided as follows:

$$\text{Fitness}(\mathbf{p}) = penalty1 + penalty2 + penalty3 + penalty4 \,. \quad \text{(S7)}$$

The penalty terms are provided below:

$$penalty1 = \begin{cases} (2.0 - AbsAmplitude)/4.0 & (AbsAmplitude < 2.0) \\ (AbsAmplitude - 6.0)/4.0 & (AbsAmplitude > 6.0) \\ 0 & (2.0 \le AbsAmplitude \le 6.0) \end{cases} , \quad \text{(S8)}$$

$$penalty2 = \begin{cases} (1.6 - RelAmplitude)/1.6 & (RelAmplitude < 1.6) \\ 0 & (RelAmplitude \ge 1.6) \end{cases} , \quad \text{(S9)}$$

$$penalty3 = \begin{cases} (23.0 - Period)/24.0 & (Period < 23.0) \\ (Period - 25.0)/24.0 & (Period > 25.0) \\ 0 & (23.0 \le Period \le 25.0) \end{cases} , \quad \text{(S10)}$$

$$penalty4 = \begin{cases} 0.1 & \text{(If an irregular oscillation occurs)} \\ 0 & \text{(If a regular oscillation occurs)} \end{cases} , \quad \text{(S11)}$$

where the absolute amplitude and period are expressed in units of nM and hour, respectively. The minimum value of the fitness is zero. The parameter vectors with the fitness of zero are solution parameter vectors, which used in the robustness and entrainment analysis.
